# Toxoplasma gondii seropositivity in patients with depressive and anxiety disorders

**DOI:** 10.1192/j.eurpsy.2021.230

**Published:** 2021-08-13

**Authors:** N. De Bles, J. Van Der Does, L. Kortbeek, A. Hofhuis, G. Van Grootheest, A. Vollaard, R. Schoevers, A. Van Hemert, B. Penninx, E. Giltay

**Affiliations:** 1 Psychiatry, LUMC, Leiden, Netherlands; 2 Centre For Disease Control The Netherlands, National Institute for Public Health and the Environment, Bilthoven, Netherlands; 3 Psychiatry, Amsterdam Public Health Research Institute, Amsterdam UMC, Vrije Universiteit, Amsterdam, Netherlands; 4 Psychiatry, UMCG, Groningen, Netherlands

**Keywords:** Toxoplasma gondii, Depression, cognitive reactivity, Anxiety

## Abstract

**Introduction:**

Toxoplasma gondii (T. gondii) is an obligate intracellular parasite that is estimated to be carried by one-third of the world population. While evidence has been found for a relationship between T. gondii infection and schizophrenia, its relationship with other psychiatric disorders like depressive and anxiety disorders shows inconsistent results.

**Objectives:**

The aim of the present study was to examine whether T. gondii seropositivity is associated with affective disorders, as well as with aggression reactivity and suicidal thoughts.

**Methods:**

In the Netherlands Study of Depression and Anxiety (NESDA), T. gondii antibodies were assessed in patients with current depressive (n=133), anxiety (n=188), comorbid depressive and anxiety (n=148), and remitted disorders (n=889), as well as in healthy controls (n=373) based on DSM-IV criteria. Seropositivity was analyzed in relation to disorder status, aggression reactivity and suicidal thoughts using multivariate analyses of covariance and regression analyses.

**Results:**

Participants were on average 51.2 years (SD = 13.2), and 64.4% were female. Seropositivity was found in 673 participants (38.9%). A strong positive association between T. gondii seropositivity and age was observed. No significant associations were found between T. gondii seropositivity and disorder status, aggression reactivity and suicidal thoughts. The adjusted odds ratio (OR) for any remitted disorder versus controls was 1.13 (95% CI: 0.87-1.49), and for any current disorder versus controls was 0.94 (95% CI: 0.69- 1.28).

**Conclusions:**

No evidence was found for a relationship between affective disorders and T. gondii infection

**Disclosure:**

No significant relationships.

